# Dantu Blood Group Erythrocytes Form Large *Plasmodium falciparum* Rosettes Less Commonly

**DOI:** 10.4269/ajtmh.23-0347

**Published:** 2024-01-30

**Authors:** Molly S. A. Carlier, Wilfred Nyamu, Johnstone Makale, Thomas N. Williams, J. Alexandra Rowe, Silvia N. Kariuki

**Affiliations:** ^1^ Institute for Immunology and Infection Research, School of Biological Sciences, University of Edinburgh, United Kingdom;; ^2^ Department of Epidemiology and Demography, KEMRI–Wellcome Trust Research Programme, Kilifi, Kenya;; ^3^ Institute of Global Health Innovation, Department of Surgery and Cancer, Imperial College London, United Kingdom

## Abstract

Dantu erythrocytes, which express a hybrid glycophorin B/A protein, are protective against severe malaria. Recent studies have shown that Dantu impairs *Plasmodium falciparum* invasion by increasing erythrocyte membrane tension, but its effects on pathological host–parasite adhesion interactions such as rosetting, the binding of uninfected erythrocytes to *P. falciparum*–infected erythrocytes, have not been investigated previously. The expression of several putative host rosetting receptors—including glycophorin A (GYPA), glycophorin C (GYPC), complement receptor 1 (CR1), and band 3, which complexes with GYPA to form the Wright^b^ blood group antigen—are altered on Dantu erythrocytes. Here, we compare receptor expression, and rosetting at both 1 hour and 48 hours after mixing with mature trophozoite-stage Kenyan laboratory–adapted *P. falciparum* strain 11019 parasites in Dantu and non-Dantu erythrocytes. Dantu erythrocytes showed lower staining for GYPA and CR1, and greater staining for band 3, as observed previously, whereas Wright^b^ and GYPC staining did not vary significantly. No significant between-genotype differences in rosetting were seen after 1 hour, but the percentage of large rosettes was significantly less in both Dantu heterozygous (mean, 16.4%; standard error of the mean [SEM], 3.2) and homozygous donors (mean, 15.4%; SEM, 1.4) compared with non-Dantu erythrocytes (mean, 32.9%; SEM, 7.1; one-way analysis of variance, *P =* 0.025) after 48 hours. We also found positive correlations between erythrocyte mean corpuscular volume (MCV), the percentage of large rosettes (Spearman’s r_s_ = 0.5970, *P =* 0.0043), and mean rosette size (r_s_ = 0.5206, *P =* 0.0155). Impaired rosetting resulting from altered erythrocyte membrane receptor expression and reduced MCV might add to the protective effect of Dantu against severe malaria.

## INTRODUCTION

*Plasmodium falciparum* causes more than 220 million clinical malaria infections annually, of which between 1% and 3% develop into severe, life-threatening disease episodes.^1^ Two key processes that are important in the pathophysiology of severe malaria are the adhesion of *P. falciparum*–infected erythrocytes to the lining of blood vessels (cytoadhesion)^2^ and to uninfected erythrocytes, a feature known as rosetting.^3^ Both lead to the obstruction of microvascular blood flow,^4^ tissue ischemia,^5^ anaerobic glycolysis,^6^ and acidosis.^7^ Both cytoadhesion and rosetting are mediated by the binding of parasite-encoded adhesion proteins on the surface of infected erythrocyte to specific host cell receptors.^8^^,^^9^ A number of erythrocyte surface molecules have been proposed as rosetting receptors,^10^ including the blood group antigens A and B,^11^ complement receptor 1 (CR1),^8^^,^^12^ glycophorin A (GYPA),^13^ and glycophorin C (GYPC).^14^ Most recently, monoclonal antibody Fab fragments against the Wright^b^ blood group antigen, formed by GYPA in complex with the erythrocyte anion transporter band 3 (AE1/SLC4A1),^15^ have been shown to disrupt rosettes,^16^ suggesting that the GYPA/band 3 complex could be a host rosetting receptor.

**Figure 1.**
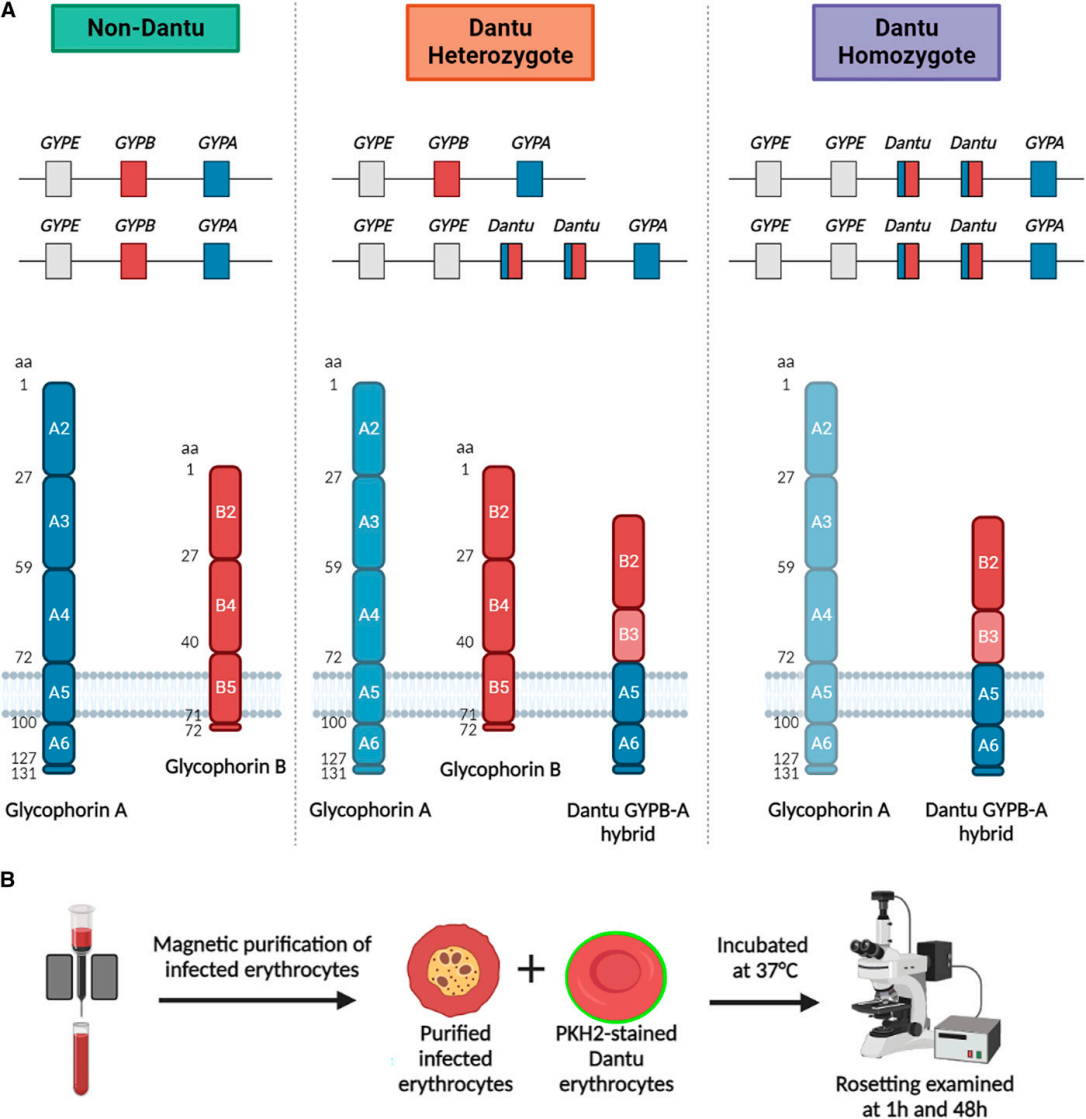
Schematic of Dantu glycophorins and the Dantu rosetting experiment. (**A**, top) Organization of non-Dantu, Dantu heterozygote, and Dantu homozygote glycophorin genes.^19^ (**A**, bottom) Composition of glycophorin A (GYPA), glycophorin B, and the Dantu glycophorin B-A hybrid (GYPB-A)across the Dantu genotypes. The regions of the glycophorin molecules encoded by the corresponding exons are numbered.^20^ The lower expression of GYPA in Dantu erythrocytes is indicated by paler shading. (**B**) Dantu rosetting experimental design in which purified *Plasmodium falciparum*–infected erythrocytes stained with ethidium bromide were mixed with Dantu homozygous, Dantu heterozygous, or non-Dantu erythrocytes stained with PKH2 fluorescent dye, with rosetting assessed after 1 hour and 48 hours.

Glycophorins are abundant, highly glycosylated erythrocyte surface proteins that are responsible for creating a negatively charged, repulsive force between erythrocytes to prevent hemagglutination.^17^ Glycophorin A is a 131-amino acid glycoprotein comprised of a total of 72-amino acid extracellular domain, a single transmembrane-spanning domain, and a 36-amino acid cytoplasmic domain.^18^ Glycophorin B (GYPB) is a smaller, less abundant glycoprotein that is closely related to GYPA, and is comprised of a total of 72 amino acids that form a short extracellular domain and transmembrane region with almost no intracellular domain. The genes that encode GYPA and GYPB (*GYPA* and *GYPB*) are adjacent on chromosome 4^18^ (Figure 1A^19^^,^^20^). This genetic region is a recombination hotspot involving complex rearrangements at the *GYPA*/*B*/*E* locus, one outcome of which is a hybrid *GYPB*/*A* gene that encodes the Dantu glycoprotein, comprising the extracellular domain of GYPB, and the transmembrane and cytoplasmic domains of GYPA (Figure 1A).

Dantu is a rare glycophorin variant that is found at a frequency of up to 9.5% on the coast of Kenya,^21^ but is generally absent outside East Africa.^18^^,^^19^ Previous genome-wide association studies have shown that Dantu is associated strongly with protection against all clinical forms of severe malaria.^18^^,^^21^ More recently, we demonstrated that membrane tension is increased in Dantu erythrocytes, and that this increased tension is associated with reduced *P. falciparum* merozoite invasion, providing a plausible explanation for the malaria protective effect of Dantu.^22^ We also showed that levels of a number of membrane proteins are altered in Dantu erythrocytes, which could potentially affect rosetting.^22^ Flow cytometry showed significantly reduced levels of GYPA, which was confirmed by quantitative massive spectrometry (19% of the non-Dantu level in Dantu-variant erythrocytes, *P* < 0.0001). However, minor changes in CR1 and GYPC suggested by flow cytometry were not confirmed in the quantitative assay. Band 3 showed significantly increased staining in Dantu cells, but significantly lower levels by quantitative mass spectrometry (49% of the non-Dantu level in Dantu-variant cells, *P* < 0.05). This discrepant result may be explained by the band 3 monoclonal antibody (mAb) used in flow cytometry having greater access to its epitope in Dantu cells as a result of the reduced level of GYPA.^22^ Other studies^23^^,^^24^ have shown that the Dantu hybrid glycophorin lacks the Wright^b^ antigen, because the extracellular region of GYPA required to form the Wright^b^ epitope with band 3^15^ is missing in the hybrid protein. To our knowledge, staining for the Wright^b^ antigen in Dantu erythrocytes has not been reported previously, and the relative expression levels of Wright^b^ in Dantu and non-Dantu erythrocytes is unknown.

The previously described changes in erythrocyte membrane proteins raise the possibility that Dantu might also lead to impaired *P. falciparum* rosetting. Various other human erythrocyte polymorphisms associated with protection against severe malaria, including CR1 deficiency,^25^ blood group O,^26^^,^^27^ Hemoglobin S,^28^ Hemoglobin C,^29^ and the Knops blood group,^30^ cause reduced parasite rosetting. Because the size, strength, and frequency of rosettes all influence the degree of microvessel blockage,^4^^,^^31^ the occurrence of fewer, smaller, and weaker rosettes when parasites infect erythrocytes with malaria protective polymorphisms may improve microvascular blood flow and protect against pathology.

We hypothesized that the altered expression of rosetting receptors in Dantu erythrocytes might impair their ability to form rosettes—a mechanism of protection against severe malaria that might add to the invasion phenotype demonstrated previously.^22^ To investigate this possibility, we examined the expression of putative rosetting receptors in Dantu and non-Dantu erythrocytes by flow cytometry, and the ability of Dantu and non-Dantu erythrocytes to form rosettes with an East African *P. falciparum* line.

## MATERIALS AND METHODS

### Study participants.

Blood samples were obtained from 21 children younger than 13 years from Kilifi County on the Indian Ocean coast of Kenya.

### Dantu sample genotyping.

Genomic DNA was extracted from whole blood using a QIAmp 96 DNA QIcube HT kit on a QIAcube HT System (QIAGEN, Manchester, United Kingdom) according to the manufacturer’s instructions. Genotypes at the Dantu marker single nucleotide polymorphism, rs186873296, were determined by a CviQI (Thermo Fisher, Waltham, MA) restriction fragment length polymorphism assay as described elsewhere.^22^ The 21 samples studied here are a subset of the 42 samples described previously.^22^

### Sample preparation.

Erythrocyte samples were purified from whole blood before cryopreserving in glycerolyte^28^ and storing in liquid nitrogen. The frozen erythrocytes were shipped to Edinburgh, where they were thawed by standard methods^32^ in sets of three (ABO blood group-matched Dantu homozygous, Dantu heterozygous, and non-Dantu erythrocytes). After thawing, erythrocytes were kept at 4°C and used within 48 hours. Sample genotypes were masked until all experiments were completed.

### Monoclonal antibody Fab fragment preparation.

The mAbs used in flow cytometry are described in Table 1. Fab fragments were generated from 100 μg of IgG2a mAbs by papain digestion (5–6 hours, 37°C) using a Pierce Fab Micro Preparation Kit (44685, Thermo Fisher Scientific, Waltham, MA) according to the manufacturer’s instructions. Immunoglobulin G1 Fab fragments were prepared by Ficin digestion (3–5 hours, 37°C) using a Pierce Mouse IgG1 Fab and F(ab′) Micro Preparation Kit (44680, Thermo Fisher Scientific) according to the manufacturer’s instructions. Purified Fab fragments were concentrated using Amicon Ultra-0.5 10-kDa MWCO Centrifugal Filter Devices (UFC501024, Sigma-Aldrich St. Louis, MO) according to the manufacturer’s instructions. Total protein in Fab preparations was quantified using a Nanodrop Spectrophotometer (Thermo Fisher Scientific), and successful digestion was confirmed by sodium dodecyl sulfate–polyacrylamide gel electrophoresis.

**Table 1:** Details of primary and secondary antibodies used

Primary antibody	Clone	Details	Secondary antibody
Secondary-only control	–	–	1:1,000 Alexa 488 conjugated goat antimouse IgG (H+L) (A-11029, Thermo Fisher Scientific)
IgG1 isotype control	MOPC-21	IgG1κ, Biolegend London, United Kingdom, 400102	
IgG2a isotype control	MOPC-173	IgG2aκ, Biolegend, 400202	
Wright^b^	BRIC 14	IgG2aκ, IBGRL Bristol, United Kingdom, 9413PA	
Band 3	BRIC 200	IgG1κ, IBGRL, 9468PA	
Glycophorin A	BRIC 256	IgG1κ, IBGRL, 9415PA	
Complement receptor 1	J3D3	IgG1, GeneTex Irvine, CA, GTX44217	
Glycophorin C	Ret40f	IgG1, GeneTex, GTX29521	

H+L = heavy and light chains; IBGRL = International Blood Group Reference Laboratory. All primary antibodies are mouse monoclonal antibodies.

### Flow cytometry analysis of erythrocyte surface receptors.

Dantu homozygous, Dantu heterozygous, and non-Dantu erythrocytes were stained for Wright^b^ (BRIC 14), GYPA (BRIC 256), band 3 (BRIC 200), GYPC (Ret40f), and CR1 (J3D3), and were analyzed by flow cytometry. A packed cell volume (PCV) (0.5 μL) of erythrocytes was added to each of eight tubes, washed once with 750 μL phosphate-buffered saline (PBS), washed once with 750 μL PBS/0.1% bovine serum albumin (BSA), and resuspended in 4 μL PBS/0.1% BSA. Erythrocytes were incubated with 0.1 mg/mL antibody Fab fragments or isotype controls in PBS/1% BSA at a final hematocrit (Ht) of 8% for 1 hour at 37°C, with mixing every 15 minutes. Samples were washed twice with 750 μL PBS/0.1% BSA and incubated at 2.5% Ht with 1:1000 dilution of Alexa Fluor 488–conjugated goat antimouse IgG in PBS/0.1% BSA for 45 minutes on ice in darkness, with mixing every 15 minutes. Erythrocytes were washed twice with cold PBS/0.1% BSA, then resuspended in cold 500 μL FACS buffer (PBS/0.5% BSA/0.02% sodium azide). Samples were run on an LSRII flow cytometer (BD Biosciences, Wokingham, United Kingdom) using the 530/30 Blue (488-nm) laser, with at least 10,000 singlet erythrocyte events counted per sample. Data were analyzed with FlowJo software (BD Biosciences), and the geometric mean fluorescence intensity of each sample was used to compare staining among genotypes. The gating strategy and background staining with isotype controls are shown in Supplemental Figures 1.

### Dantu erythrocyte staining with PKH2 for rosetting assays.

Each set of Dantu homozygous, Dantu heterozygous, and non-Dantu erythrocytes were counted with a hemocytometer, and a 50-μL PCV (5 × 10^8^ erythrocytes) was stained with PKH2 green-fluorescent cell membrane dye (PKH2GL, Sigma-Aldrich). Erythrocytes were washed once in 5% D-glucose and resuspended in 500 μL D-glucose. One microliter of PKH2 in 500 μL D-glucose was added to the erythrocyte suspension, which was then mixed gently and continuously for 2 minutes at room temperature. Four hundred microliters FBS (Thermo Fisher Scientific) was added to quench the dye, and erythrocytes were washed three times with incomplete RPMI 1640 (BE12-167F, Lonza, Manchester, United Kingdom; supplemented with 25 mM HEPES [Sigma-Aldrich], 2 mM glutamine [Thermo Fisher Scientific], 16 mM glucose, and 25 μg/mL gentamicin [Lonza]) and resuspended at 50% Ht in incomplete RPMI.

### Parasites and parasite culture.

Kenyan laboratory–adapted *P. falciparum* strain 11019^33^ was cultured at 2% Ht in O+ erythrocytes (Scottish National Blood Transfusion Service, Edinburgh, United Kingdom) and complete RPMI 1640 (as described earlier for incomplete RPMI, with an additional 0.25% Albumax II [Thermo Fisher Scientific] and 5% AB pooled human serum [CR300, TCS Biosciences Botolph Claydon, United Kingdom]). Cultures were gassed with 1% oxygen (O_2_)/5% carbon dioxide (CO_2_)/94% nitrogen (N_2_) and incubated at 37°C. Parasitemia was assessed by 10% Giemsa-stained thin blood smear.

### Purification of infected erythrocytes.

Infected erythrocyte purification and rosetting assays were performed either on the day of PKH2 staining or the following day, depending on the availability of mature-stage parasitized erythrocytes. *Plasmodium falciparum*–infected erythrocytes were purified using a magnetic-activated cell sorting (MACS) column (Miltenyi Biotec Bisley, United Kingdom) as described previously,^34^ with the addition of 1 mg/mL heparin to all buffers to disrupt rosettes. The postpurification parasitemia was determined by staining an aliquot of erythrocyte suspension with 25 μg/mL ethidium bromide (37°C for 2 minutes) and by assessing the percentage of infected erythrocytes out of 200 erythrocytes counted on a wet preparation by fluorescence microscopy. This ranged from 58% to 73%, depending on the efficacy of the MACS purification.

### Purified, infected erythrocyte mixing with PKH2-stained Dantu erythrocytes.

Approximately 20 μL PCV of the purified, infected erythrocyte pellet was resuspended in 150 μL of incomplete RPMI/1 mg/mL heparin/0.5% BSA, and 50-μL aliquots were placed into three Eppendorf tubes. One hundred microliters of 50% Ht PKH2-stained erythrocytes from each donor was centrifuged at 1,000 × *g* for 2 minutes, the supernatant removed, and the erythrocytes resuspended in incomplete RPMI/1 mg/mL heparin/0.5% BSA. The stained erythrocyte suspensions were added to the tubes containing the purified, infected erythrocytes, giving a final parasitemia of ∼4%. Cells were washed three times with incomplete RPMI, once with complete RPMI, and resuspended in 2.5 mL complete RPMI. The erythrocyte suspension was transferred to a T25 culture flask and gassed for 30 seconds with 1% O_2_/5% CO_2_/94% N_2_ and incubated at 37°C.

### Rosetting assays.

Rosetting assays were performed 1 hour and 48 hours after mixing purified, infected erythrocytes with stained Dantu erythrocytes (Figure 1B). A 200-μL aliquot of culture suspension was stained with 25 μg/mL ethidium bromide for 2 minutes at 37°C. A 10-μL aliquot of this stained erythrocyte suspension was placed on a microscope slide and covered with a 22- × 22-mm coverslip. The erythrocytes were viewed with a Leica DM2000 fluorescent microscope (×40 magnification), with white light combined with the TRITC filter to identify ethidium bromide–stained infected erythrocytes or the FITC filter to view the PKH2 staining. The rosette frequency of each sample was determined by counting the percentage of mature, infected (pigmented trophozoite- or schizont-infected) erythrocytes binding two or more uninfected erythrocytes, at least one of which had to be PKH2 stained. This allowed for the determination of rosetting with the test erythrocytes, while excluding rosettes formed with the subpopulation of unstained, uninfected erythrocytes carried over in the MACS purification. One hundred infected erythrocytes were assessed for rosetting in two different parts of the wet preparation slide, and the values were averaged to give the rosette frequency based on 200 infected erythrocytes per sample. Mean rosette size was determined by counting the number of uninfected erythrocytes that were bound to infected erythrocytes in ∼50 rosettes per sample. Only rosettes that contained PKH2-stained erythrocytes were included in this count. The percentage of large rosettes was determined from the rosette size counts, with a large rosette defined as four or more uninfected erythrocytes per rosette. This definition was based on studies of rosette size and stability showing enhanced survival of large rosettes in narrow vessels.^31^

### Measurement of erythrocyte mean corpuscular volume.

Erythrocyte mean corpuscular volume (MCV) was measured using an automated cell Coulter counter (Beckman Coulter, Indianapolis, IN) as described previously.^22^

### Statistical analysis and graphing.

Data were visualized and analyzed using GraphPad Prism v7.0 (GraphPad Software La Jolla, CA). Differences between sample means were analyzed by one-way analysis of variance (ANOVA), with Dunnett’s multiple comparisons test to compare values in Dantu homozygous and Dantu heterozygous samples to those in non-Dantu erythrocytes. The χ^2^ test was used to examine differences between genotypes in the frequency distribution of rosette size. Spearman’s rank correlation coefficient was used to assess the relationship between erythrocyte MCV and rosetting. All raw data are provided in Supplemental datasheets S1 through S3.

## RESULTS

### Dantu erythrocytes express putative rosetting receptors including the Wright^b^ antigen.

**Figure 2.**
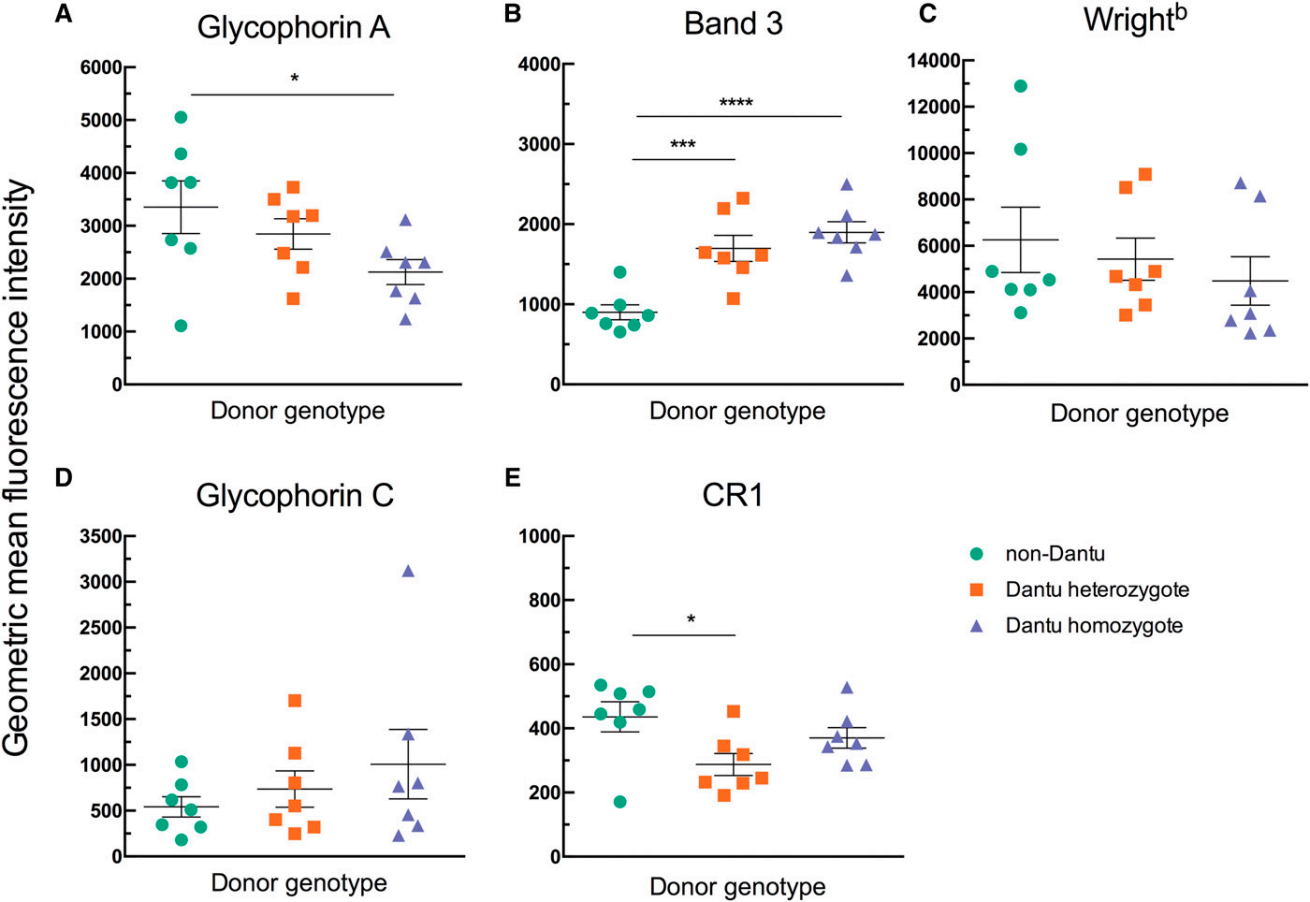
Staining for erythrocyte rosetting receptors varies with Dantu genotype. The relative expression of putative erythrocyte rosetting receptors was measured using monoclonal antibody Fab fragments and Alexa Fluor 488–conjugated secondary antibodies in flow cytometry. Seven non-Dantu, seven Dantu heterozygous, and seven Dantu homozygous samples were tested. Statistical comparison across groups was performed by one-way analysis of variance with Dunnett’s multiple comparisons test. Statistically significant differences were observed for glycophorin A (**A**) (non-Dantu versus Dantu homozygous, *P < 0.05), band 3 (**B**) (non-Dantu versus Dantu heterozygous, ***P <0.001; non-Dantu versus Dantu homozygous, ****P <0.0001), and complement receptor 1 (CR1) (**E**) (non-Dantu versus Dantu heterozygous, *P <0.05), but not for Wright^b^ (**C**) (*P =* 0.5572) or glycophorin C (**D**) (*P =* 0.4472). Bars show the mean and standard error of the mean for each genotype.

Immunofluorescent staining was carried out to assess the relative expression levels of erythrocyte rosetting receptors in Dantu homozygous, Dantu heterozygous, and non-Dantu erythrocytes. Glycophorin A staining was less in Dantu homozygous erythrocytes compared with non-Dantu erythrocytes, and band 3 staining was greater, confirming previous results^22^ (Figure 2 and Supplemental Figure S2). Staining for CR1 was significantly less in Dantu heterozygotes, again confirming previous data,^22^ whereas GYPC staining showed no difference among genotypes. Staining for the Wright^b^ antigen tended to mirror the pattern of GYPA staining, but the variation within genotypes was large and no statistically significant differences were observed (Figure 2). Given the requirement for the extracellular region of GYPA to bind to band 3 to create the Wright^b^ epitope,^15^ a correlation between GYPA and Wright^b^ staining would be expected. Examination of the flow cytometry data showed a strong positive correlation between the geometric mean fluorescent intensity for GYPA and Wright^b^ for the data set as a whole (r_s_ = 0.7373, *P =* 0.0001, Supplemental Figure S3). Each genotype also showed a positive correlation for GYPA and Wright^b^ staining, although the strength of the relationship varied, being most marked in the non-Dantu donors (non-Dantu r_s_ = 0.9643, *P =* 0.0028; Dantu heterozygous r_s_ = 0.7143, *P =* 0.0881; Dantu homozygous r_s_ = 0.5225, *P =* 0.2397).

### Large rosettes are less common in Dantu erythrocytes.

**Figure 3.**
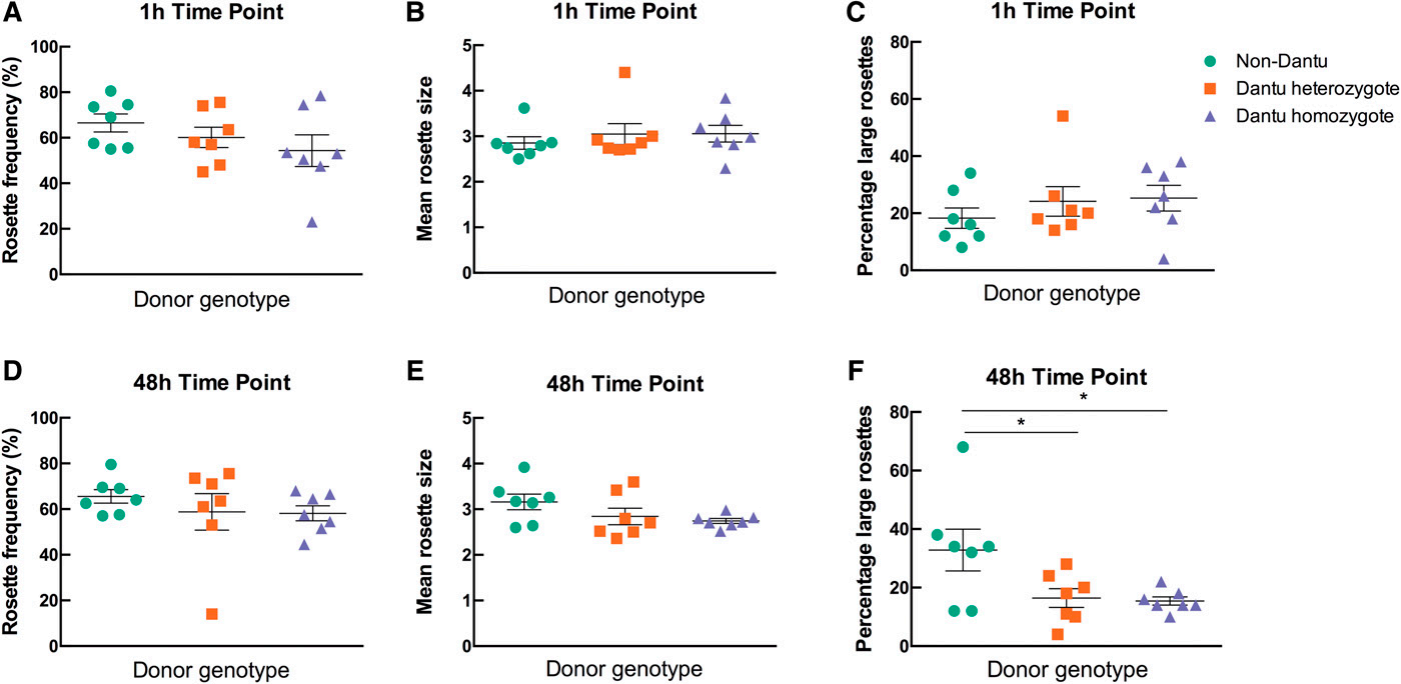
Fewer large rosettes observed in Dantu erythrocytes. The ability of different Dantu genotype erythrocytes to form rosettes was measured by mixing *Plasmodium falciparum* strain 11019 purified, infected erythrocytes with PKH2-stained Dantu or non-Dantu erythrocytes. Rosetting was assessed by fluorescence microscopy after 1 hour (**A–C**) and 48 hours (**D–F**). The rosette frequency is the percentage of mature, infected (pigmented trophozoite- or schizont-infected) erythrocytes binding two or more uninfected erythrocytes, at least one of which was stained with PKH2 dye. The mean rosette size is the number of uninfected erythrocytes per rosette based on ∼50 rosettes per sample. Large rosettes are defined as those containing four or more uninfected erythrocytes. Seven non-Dantu, seven Dantu heterozygous, and seven Dantu homozygous samples were tested. Statistical comparison across groups was performed by one-way analysis of variance with Dunnett’s multiple comparisons test (*P < 0.05). Bars show the mean and standard error of the mean for each genotype.

**Figure 4.**
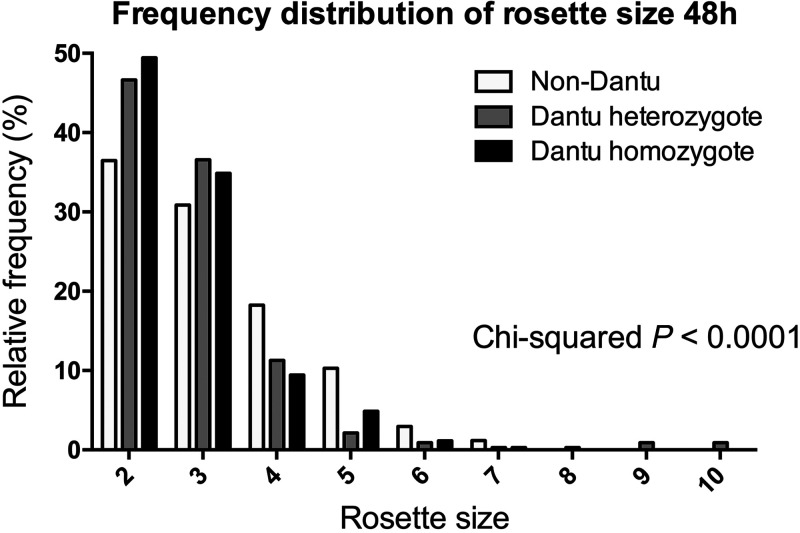
Frequency distribution of rosette size at 48 hours differs among genotypes. The rosette size data for donors within each genotype were pooled, and the relative frequency of rosettes in each size category (expressed as a percentage of all rosettes) is shown for Dantu homozygous, Dantu heterozygous, and non-Dantu donors. Rosette size indicates the number of uninfected erythrocytes in each rosette. Statistical comparison between groups was performed using a Chi-squared test, *P* < 0.0001.

To investigate whether the Dantu polymorphism influences rosetting, Dantu homozygous, Dantu heterozygous, and non-Dantu erythrocytes were stained with the PKH2 fluorescent dye then incubated with MACS-purified, *P. falciparum*–infected erythrocytes from Kenyan parasite line 11019, and rosetting was assessed at 1 hour and 48 hours. The two time points allowed for assessment of rosetting when only the uninfected erythrocytes in the suspension were stained (at 1 hour), and when the parasites had reinvaded so that both infected and uninfected erythrocytes were stained (at 48 hours). The second time point allows adhesion interactions to reach their maximum strength^35^ and also allows for any reduction in adhesion due to impaired P. falciparum erythrocyte membrane protein one (PfEMP1) display to become manifest.^29^ At the 1-hour time point, there was no significant difference in rosette frequency, mean rosette size, or percentage of large rosettes between the Dantu and non-Dantu erythrocytes (Figure 3A–C). At 48 hours, there was also no significant difference among genotypes in rosette frequency, but there was a nonsignificant trend toward smaller mean rosette size and lower variance in Dantu homozygous compared with non-Dantu erythrocytes (Figure 3D–F). The percentage of large rosettes was significantly less in Dantu homozygous donors (mean, 15.4; standard error of the mean [SEM], 1.43) and Dantu heterozygous donors (mean, 16.43; SEM 3.20) compared with non-Dantu erythrocytes at 48 hours (mean, 32.86; SEM 7.12; *P =* 0.025 one way ANOVA) (Figure 3D–F). This difference in rosette size between genotypes was also seen by examining the frequency distribution of rosette size at 48 hours, with more frequent two- and three-uninfected erythrocyte rosettes, and fewer rosettes with four, five, six, or seven uninfected erythrocytes in Dantu compared with non-Dantu donors (Figure 4; *P <*0.0001, χ^2^ test).

### There is a positive correlation between erythrocyte mean corpuscular volume and rosetting.

**Figure 5.**
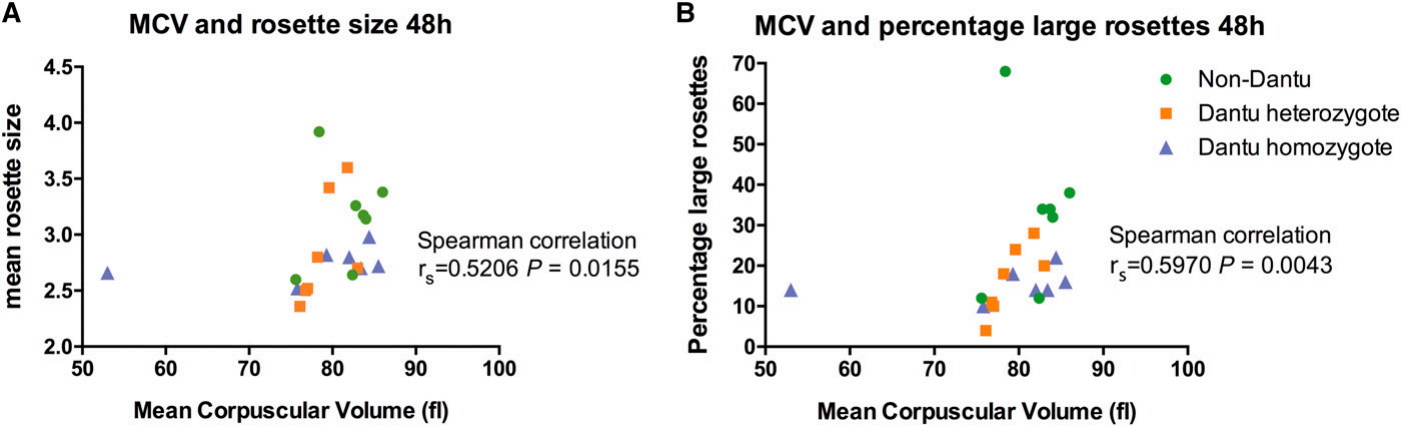
Positive correlation between mean corpuscular volume (MCV) and rosetting. The correlation between erythrocyte MCV and mean rosette size (**A**), and erythrocyte MCV and percentage of large rosettes (**B**), was determined using Spearman’s rank correlation coefficient.

On average, Dantu erythrocytes are smaller than non-Dantu erythrocytes,^22^ so we investigated whether the effect of Dantu on rosetting that we observed might be related to the reduced size of Dantu erythrocytes. We found no significant relationships between MCV and rosetting at 1 hour (Supplemental Figure S4); however, we did find a significant positive correlation between MCV and mean rosette size (Spearman’s r_s_ = 0.5206, *P =* 0.0155), and between MCV and the percentage of large rosettes (r_s_ = 0.5970, *P =* 0.0043) at 48 hours (Figure 5). The MCV values for four of seven Dantu homozygous donors were within the normal range (> 80 fL); however, their percentage of large rosettes was generally less than the non-Dantu donors with similar MCVs. This may suggest that both erythrocyte size and reduced expression of membrane receptors contribute to impaired rosetting in Dantu erythrocytes.

## DISCUSSION

In our study, we confirmed the alterations in erythrocyte membrane receptor expression in Dantu erythrocytes described previously,^22^ and investigated whether Dantu erythrocytes show any difference in rosetting compared with non-Dantu controls. We found no effect of Dantu genotype on rosette frequency (the proportion of infected erythrocytes forming rosettes), but significantly fewer large rosettes were found after the parasites had reinvaded and grown in Dantu erythrocytes for 48 hours. This result has pathophysiological implications, because large rosettes are more stable and resistant to disruption in microvessels compared with small rosettes.^31^ Hence, large rosettes are more likely to contribute to microvascular obstruction in severe malaria. The effect of Dantu on the reduction of large rosettes mirrors that of the ABO blood group, in which reduced numbers of large rosettes in group O erythrocytes compared with non-O blood groups may contribute to malaria protection.^11^^,^^26^

Our study was limited in terms of the number of erythrocyte donors tested (seven of each genotype) with a single *P. falciparum* line. Future studies using additional donors are needed to confirm the findings reported here. Furthermore, given the complexity in molecular mechanisms of rosetting, which involves diverse parasite ligands and host erythrocyte receptors,^8^^–^^14^ there is a need to carry out Dantu blood group rosetting experiments with a wider set of *P. falciparum* lines with differing rosetting phenotypes.

If the results shown here are confirmed, we can consider the possible mechanisms that could be responsible for the paucity of large rosettes in Dantu erythrocytes. These include reduced numbers of erythrocyte rosetting receptors,^5^^,^^8^ reduced expression of the parasite adhesion molecule PfEMP1^28^^,^^29^ or reduced erythrocyte size.^5^ Our data do show reduced expression of some putative erythrocyte rosetting receptors, although it is unclear whether the relatively modest changes in expression would be sufficient to impact adhesion. We did not measure PfEMP1 expression level after parasite invasion into Dantu erythrocytes, but this could be done in future studies by generating antibodies against the rosette-mediating PfEMP1 variant from the 11019 *P. falciparum* line and by testing other rosetting parasite lines^28^ for which PfEMP1 antibodies are available. Previous data show that Dantu erythrocytes have significantly lower MCV compared with non-Dantu controls.^22^ A strong positive correlation between erythrocyte MCV and mean rosette size was noted in a previous study,^36^ with microcytic erythrocytes from donors with iron-deficiency anemia and thalassaemia showing reduced rosetting capacity. In our study, we confirmed a significant positive correlation between MCV and mean rosette size, and also showed a significant positive correlation between MCV and the percentage of large rosettes. With Dantu erythrocytes on average having lower MCV, it is plausible that the size of Dantu erythrocytes is responsible in part for the impaired formation of large rosettes, combined with the specific erythrocyte membrane receptor changes, as we hypothesized originally.

One surprising finding from our study was that the Wright^b^ blood group antigen, formed by a physical association between band 3 and GYPA,^15^ was detected at high levels in all genotypes. Previous studies^23^^,^^24^ have found that the Wright^b^ antigen is not present on the hybrid glycophorin of Dantu-positive erythrocytes. This is expected because the extracellular region of GYPA, which is missing from the hybrid protein, is required for Wright^b^ expression.^15^ Why then, did we observe robust staining for the Wright^b^ antigen on Dantu homozygous erythrocytes? Recent whole-genome sequencing has confirmed that the Dantu locus contains an intact copy of the normal *GYPA* gene, in addition to the genes encoding the hybrid protein.^18^^,^^19^ Hence, although the hybrid glycophorin cannot form the Wright^b^ antigen with band 3, normal GYPA should also be present in Dantu erythrocytes to allow formation of Wright^b^. Our data show definitively that Dantu erythrocytes from both heterozygous and homozygous donors express the Wright^b^ antigen. There was a trend toward lower geometric mean fluorescence intensity for Wright^b^ staining in Dantu erythrocytes, but statistically significant differences were not detected in this small sample set. Future studies with larger sample sizes are needed to determine whether there are consistent differences in Wright^b^ expression between Dantu genotypes.

## CONCLUSION

Overall, we report that Dantu erythrocytes show reduced expression of some candidate rosetting receptors, and that the Dantu phenotype impairs the ability of *P. falciparum* to form large rosettes. These data suggest that in addition to the effects on erythrocyte membrane tension and parasite invasion shown previously, the protection against severe malaria afforded by the Dantu blood group may also include an effect on *P. falciparum* rosetting.

## Supplemental Materials

10.4269/ajtmh.23-0347 Supplemental Materials
