# Supplementary material for: Dantu Blood Group Erythrocytes Form Large Plasmodium falciparum Rosettes Less Commonly
Source: Am J Trop Med Hyg. 2024 Jan 30;110(3):436–43. doi: 10.4269/ajtmh.23-0347 (PMC10919173; doi:10.4269/ajtmh.23-0347)
Supplement: Supplemental Materials [file tpmd230347.SD1.pdf]

Supplementary figures for Carlier et al, Large *Plasmodium falciparum* rosettes are less common in Dantu erythrocytes.

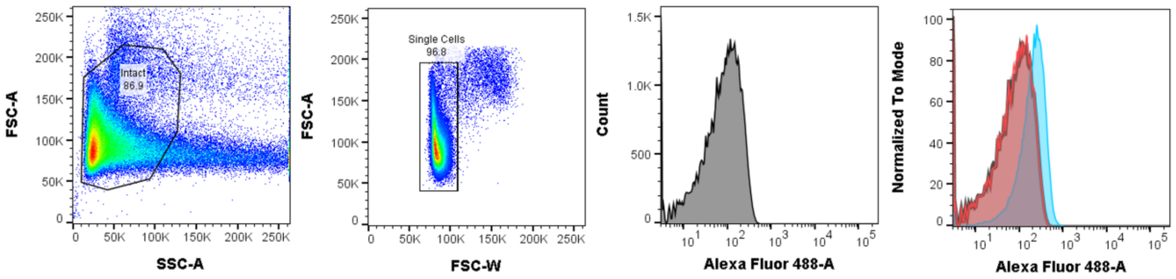

Supplementary Figure 1. Flow cytometry gating strategy used to analyse erythrocytes.

SSC-A and FSC-A were used to identify intact cells while FSC-W and FSC-A were used to identify single cells. Alexa Fluor 488 was used to identify binding of Fabs to erythrocyte surface receptors. The low level of background fluorescence seen with non-Dantu erythrocytes treated with the secondary antibody only is shown in the grey histogram. The rightmost panel shows non-Dantu erythrocytes treated with secondary antibody only (grey), IgG1 control Fab (blue) or IgG2 control Fab (red). The staining with the IgG2 isotype control was equivalent to the "secondary antibody only" control, whereas the IgG1 isotype control (blue) showed a low level of non-specific binding.

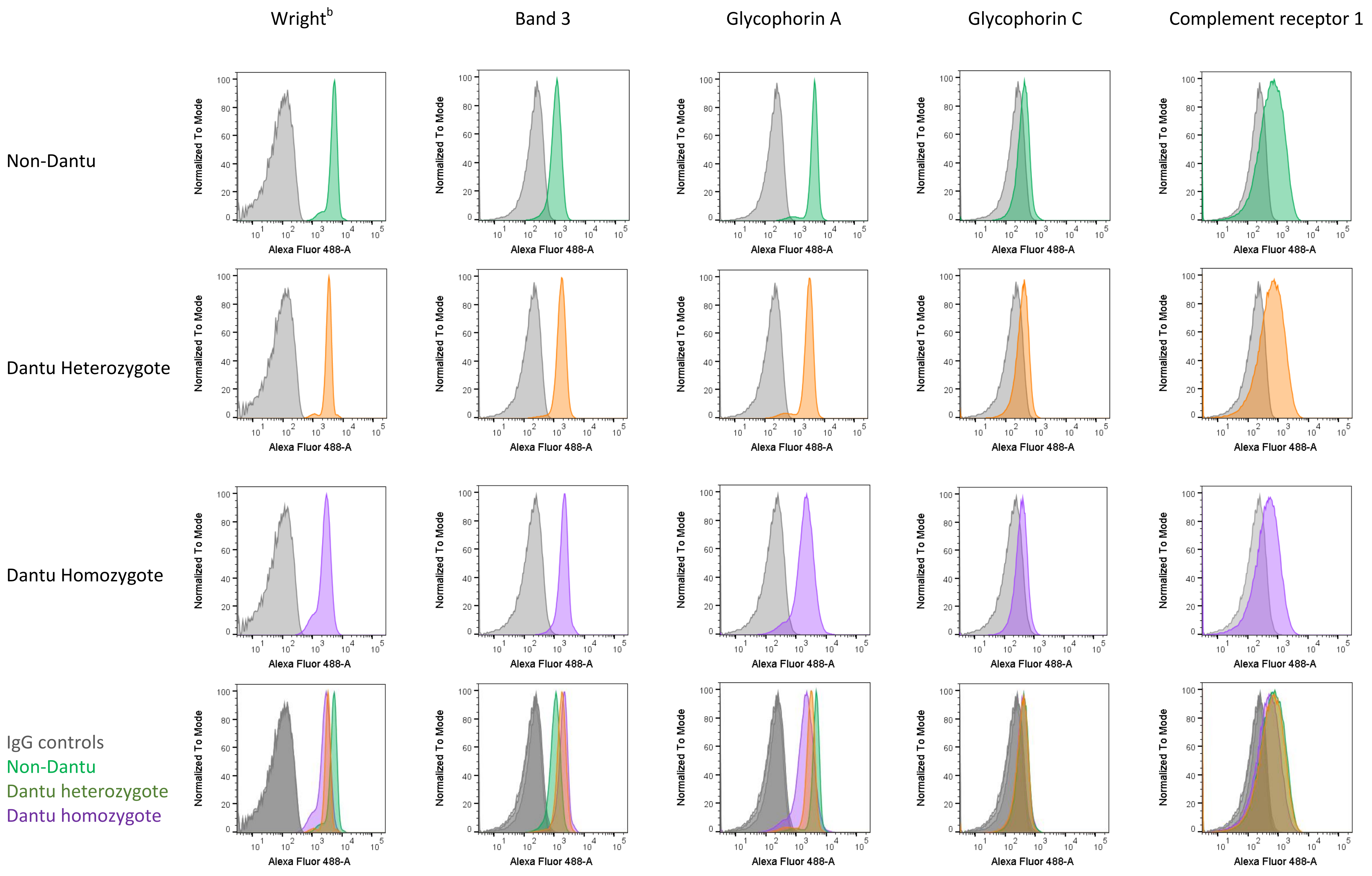

Supplementary Figure 2. Example histograms from flow cytometry staining of Dantu and non-Dantu erythrocytes. Grey = isotype control. Receptor expression is colour coded according to Dantu status; Green = non-Dantu, orange = Dantu Heterozygote, purple = Dantu Homozygote.

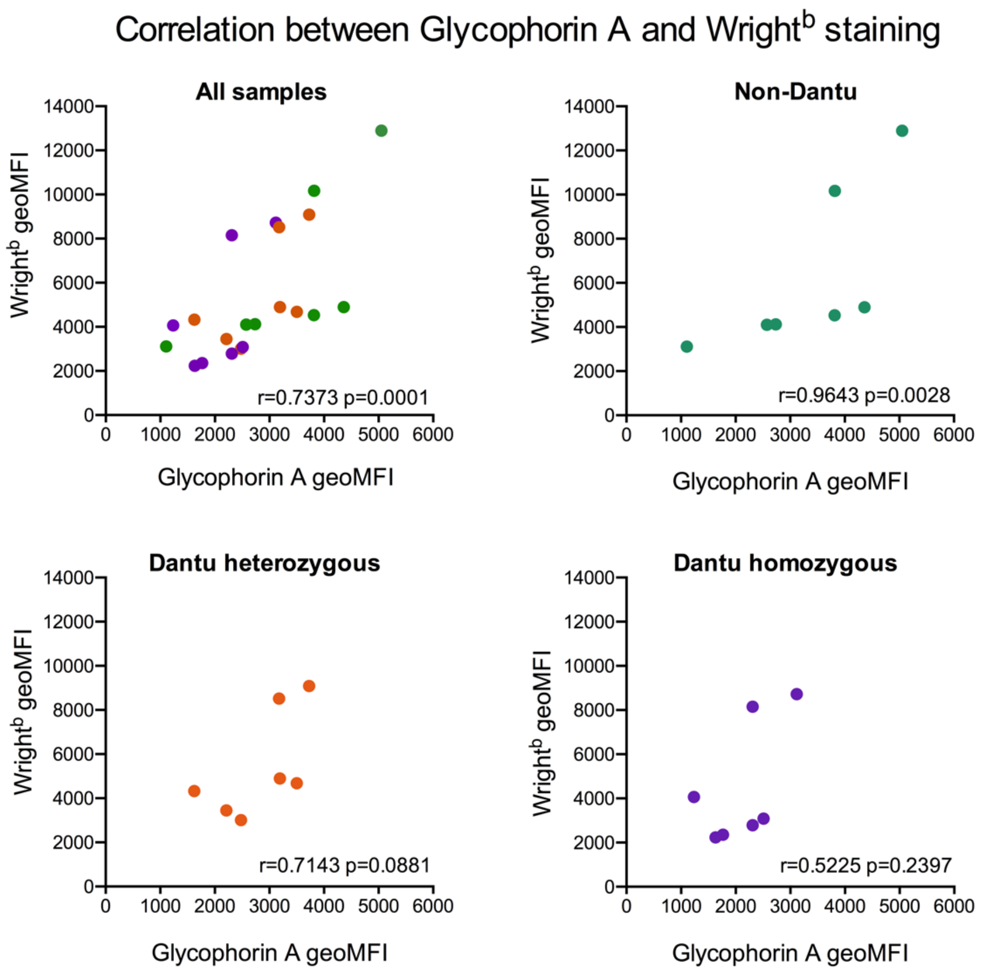

Supplementary Figure 3. Correlation between the geometric mean fluorescence intensities (MFI) for GYPA and Wright^b. The correlation between the geometric MFI for GYPA and Wright^b was determined using Spearman's rank correlation coefficient.

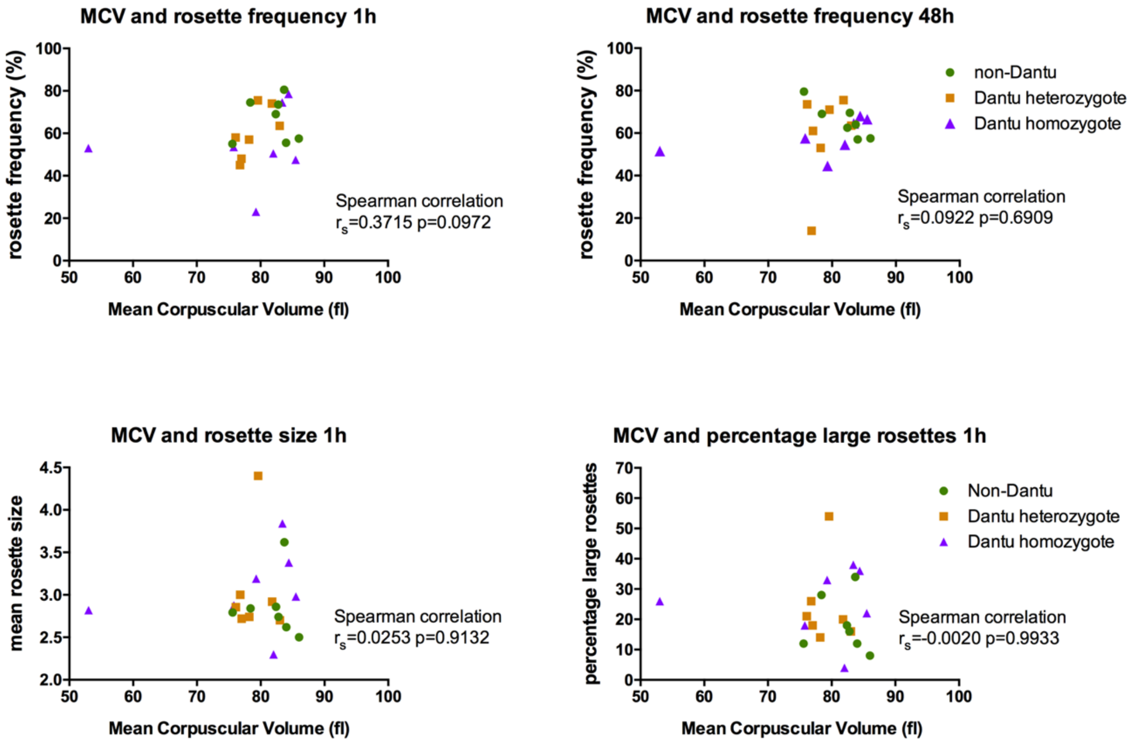

Supplementary Figure 4. Correlation between MCV and rosetting. The correlation between erythrocyte MCV and rosette frequency, mean rosette size and percentage large rosettes after 1h, and MCV and rosette frequency after 48h were determined using Spearman's rank correlation coefficient.
